# Severe psychosocial deprivation in early childhood is associated with increased DNA methylation across a region spanning the transcription start site of *CYP2E1*

**DOI:** 10.1038/tp.2016.95

**Published:** 2016-06-07

**Authors:** R Kumsta, S J Marzi, J Viana, E L Dempster, B Crawford, M Rutter, J Mill, E J S Sonuga-Barke

**Affiliations:** 1 Department of Genetic Psychology, Faculty of Psychology, Ruhr-University Bochum, Bochum, Germany; 2 MRC Social, Genetic and Developmental Psychiatry Centre, Institute of Psychiatry, Psychology and Neuroscience, King's College London, London, UK; 3 University of Exeter Medical School, University of Exeter, Exeter, UK; 4 Department of Psychology, Institute for Disorders of Impulse and Attention, Developmental Brain-Behavior Laboratory, University of Southampton, Southampton, UK; 5 Department of Experimental Clinical and Health Psychology, Ghent University, Ghent, Belgium

## Abstract

Exposure to adverse rearing environments including institutional deprivation and severe childhood abuse is associated with an increased risk for mental and physical health problems across the lifespan. Although the mechanisms mediating these effects are not known, recent work in rodent models suggests that epigenetic processes may be involved. We studied the impact of severe early-life adversity on epigenetic variation in a sample of adolescents adopted from the severely depriving orphanages of the Romanian communist era in the 1980s. We quantified buccal cell DNA methylation at ~400 000 sites across the genome in Romanian adoptees exposed to either extended (6–43 months; *n*=16) or limited duration (<6 months; *n*=17) of severe early-life deprivation, in addition to a matched sample of UK adoptees (*n*=16) not exposed to severe deprivation. Although no probe-wise differences remained significant after controlling for the number of probes tested, we identified an exposure-associated differentially methylated region (DMR) spanning nine sequential CpG sites in the promoter-regulatory region of the cytochrome P450 2E1 gene (*CYP2E1*) on chromosome 10 (corrected *P*=2.98 × 10^−5^). Elevated DNA methylation across this region was also associated with deprivation-related clinical markers of impaired social cognition. Our data suggest that environmental insults of sufficient biological impact during early development are associated with long-lasting epigenetic changes, potentially reflecting a biological mechanism linking the effects of early-life adversity to cognitive and neurobiological phenotypes.

## Introduction

Brain circuits underpinning cognition and socioemotional functioning are sculpted by social experiences during early life.^1^ Deficient or adverse social environments during this period can increase long-term vulnerability for psychiatric disorders.^2^ Understanding the mechanisms linking negative experiences to chronic mental health effects is a key target for translational developmental neurobiology.^3^ One hypothesis is that severe social adversity induces long-term alterations to gene expression and function through dynamic epigenetic modifications.^4^ Experimental studies in model organisms, for example, have shown that variation in maternal behavior brings about epigenetic alterations and associated changes in gene expression at specific loci, underlying life-long phenotypic differences in physiology and behavior, including neuroendocrine stress responsiveness, fear-related behavior and attentional processes, cognitive development, female reproductive behavior and maternal care itself (see Zhang and Meaney^5^ for a review). There is some evidence for similar epigenetic alterations in response to various environmental stressors in humans, including prenatal exposure to famine,^6^ psychosocial stress during infancy and pre-school years,^7^ early-life socioeconomic status,^8^ and childhood abuse.^4, 9, 10, 11^ However, direct replication of the effects observed in experimental animal models in humans remains challenging for a number of reasons. Most sample cohorts are characterised by considerable heterogeneity in the nature, timing and severity of adverse exposures, and there is considerable confounding between early and continuing later adversity and between adversity and consequent mental health problems.

Because of necessary ethical constraints, ‘natural experiments'—that is, studies in which exposure to severe adversity is not under direct experimental control—are the best available method for examining epigenetic changes following exposure to severe environmental conditions in human populations.^12^ The English and Romanian Adoptees study (ERA) is a prospective longitudinal study of the effects of severe adversity experienced by children before the age of 43 months in grossly depriving Romanian orphanages before they were adopted into UK families at the fall of the Ceauşescu regime in 1989.^13^ The children were followed across development and have been assessed at ages 4, 6, 11 and 15 years, with follow-up data collection at the age of 23 years just completed. ERA represents a powerful ‘natural experiment' to test the epigenetic hypothesis of the effects of psychosocial adversity. This is because the ERA children (i) were typically exposed to severe deprivation from just after birth for variable, but defined, periods of time (2 weeks to 43 months) and (ii) then experienced a sudden, precisely timed, radical change from a profoundly depriving environment to a nurturing adoptive family one. Furthermore, whereas many in the cohort have displayed long-term persistent deprivation-related problems, at least to adolescence, other adoptees are highly resilient, being indistinguishable in terms of mental health compared with their non-deprived peers. There is a strong association between length of institutional deprivation and risk for persisting deficits. Individuals adopted before 6 months were found to have rates of impairment no different from non-exposed populations, whereas about half of the samples adopted between the ages of 6 and 43 months showed continuing psychological deficits to adolescence.^14^ Within the ERA cohort, early adversity is associated with both intellectual and social behavioral deficits, with a characteristic pattern of social impairment across two domains. The first has been termed quasi-autism and is a behavioral pattern characterized by autistic-like features, particularly abnormal preoccupations and intense circumscribed interests. The difference to classical autism lies in greater, albeit unusual, social interest and flexibility, and in the diminishing intensity of these features over time.^15^ Deficits in Theory of Mind (ToM) provide substantial mediation of the quasi-autistic pattern.^16, 17^ The second shares many features with the new DSM diagnostic category ‘Disinhibited Social Engagement Disorder'^18^ and is characterized by a marked disregard for social boundaries, inappropriate levels of familiarity, social disinhibition and self-disclosure.^15, 19^ Across all ages there is a substantial overlap between quasi-autism and disinhibited social engagement.^15^ These core deficits in social cognition and behavior are often accompanied by cognitive deficits (at the age of 15, the mean intelligence quotient (IQ) of the late adopted group was one s.d. below the UK and early adopted group) and symptoms of attention-deficit hyperactivity disorder.^20^

The persistent nature of the negative impact of early severe deprivation, which for many in the sample was not eradicated by positive post-adoption experiences, is consistent with an enduring biological impact of early deprivation. In this study we aimed to test whether exposure to extreme deprivation is associated with altered DNA methylation among Romanian adoptees and whether these effects are also related to variation in intellectual and social functioning in the ERA group.^17^

## Materials and methods

### Sample

The ERA sample was drawn from children adopted from Romania into families residing in England between February 1990 and September 1992, who were aged 43 months, or below, at the time of entry to the United Kingdom (see Rutter *et al.*^13^ for detailed description of historical background and sample characteristics). Briefly, following an age-stratified sampling design, the ERA study enrolled roughly equal numbers of children adopted before 6 months, between 6 and 24 months and over 24–43 months. The Romanian children were compared with a group of 52 children born and adopted within the United Kingdom before the age of 6 months. None of the children in the within-UK adoptee group had been exposed to early deprivation, neglect or abuse. Most children had been placed in institutions in the first weeks of life (the mean age of entry was 0.34 months, s.d.=1.26, making it unlikely that the reason for their admission into institutions was manifest handicap). Out of 217 subjects, DNA samples were available for 131 individuals, and 49 individuals were selected for the present study. Our selection strategy was based on the finding that at 11 and 15 years of follow-up there was a step-wise relationship between length of institutional rearing and risk for psychosocial and developmental outcome, with the difference laying between institutional deprivation that did not continue beyond the age of 6 months and institutional deprivation that persisted longer than that.^13^ Accordingly, for the current analyses we focused on the comparison between individuals experiencing extended (more than 6 months; *n*=16) or limited (less than 6 months; *n*=17) deprivation. Furthermore, these two groups were compared with a subgroup of individuals from the within-UK adoptee group (*n*=16). Selection of the participants was carried out at random for the UK comparison group and the group experiencing <6-month deprivation. For the >6-month category, participants were selected at random from the subgroup of individuals showing deprivation-related impairments. As shown in Supplementary Table 1 and Supplementary Table 2, there were no significant differences between groups in gender, smoking or the abuse of alcohol, cannabis or other drugs. With the exception of one individual in the >6-month exposure group using antidepressants at time of sampling, and the elevated rate of methylphenidate use in the >6-month exposure group, there was no use of medication among the samples included in this study (antipsychotics, mood stabilizers and antidepressants). Furthermore, there were no significant differences in birth weight between the early and late adopted Romanian adoptees. As shown in Supplementary Table 1, and comparable to the ERA sample as a whole,^16^ deficits in ToM and lower IQ were observed in the group with extended deprivation. Furthermore, as previously observed,^13^ there were no differences between the short length of deprivation and the UK comparison group. The study was approved by the King's College London ethics committee. Parents gave informed consent for themselves and their children.

### DNA methylation profiling

Buccal cell samples were collected at the age of 15 and DNA isolated using a standard method.^21^ Genomic DNA was treated with sodium bisulfite in duplicate using the EZ-96 DNA methylation-gold kit (Zymo Research, Irvine, CA, USA) and DNA methylation profiled using the Infinium HumanMethylation450 BeadChip (Illumina, San Diego, CA, USA) processed on an Illumina HiScan System (Illumina) according to the manufacturers' standard protocol. All samples were randomized within and between arrays to avoid potential batch effects.

### Data-processing and quality control

Signal intensities for each probe were extracted using the Illumina GenomeStudio software and were imported into R (ref. 22) using the *methylumi* ^23^ and *minfi* package.^24^ Multidimensional scaling plots of variable probes on the sex chromosomes were used to check that the predicted gender corresponded with the reported gender for each individual. Stringent quality-control checks, quantile normalization and separate background adjustment of methylated and unmethylated intensities of type I and II probes were implemented using the *wateRmelon* package in R.^25^ Samples with ⩽5% of sites with a detection *P*-value>0.05 were included in subsequent analyses, and probes with >5% of samples with a detection *P*-value>0.05 or a bead count <3 in 5% of samples were removed. We excluded the 65 single-nucleotide polymorphism probes, probes on sex chromosomes, cross-hybridizing probes^26^ and probes with common (minor allele frequency>5%) single-nucleotide polymorphisms in the CG or single-base extension position from subsequent analysis, with the final analysis data set comprising 382 291 probes.

### Cognitive and sociocognitive abilities

Cognitive abilities were assessed with the short form of the Wechsler Intelligence Scale for Children (WISC III, UK) at 15 years of follow-up. This is the most commonly used standardized measure of young people's cognitive abilities, and it has established reliability.^27^ Four subscales of the WISC were employed: two from the verbal scales (vocabulary and similarities) and two from the performance scales (block design and object assembly). These four subscales were selected to provide a good estimate of full-scale IQ (reliability coefficient=0.94).^28^ The four subscales were pro-rated to form a full-scale IQ for subsequent analyses. At the age of 11 years, the ‘strange stories' task^29^ was employed as a measure of ToM. The task required the children to respond to seven ToM-related vignettes. The responses to the vignettes were scored in terms of the level of ToM understanding displayed, with ‘0' indicating a non-ToM-related response, ‘1' indicating basic-level ToM understanding and ‘2' indicating evidence of more sophisticated ToM understanding. Scores were combined across the seven stories, and the mean scores were used in analyses.

### Data analysis

Data was analyzed using a *t*-test for group mean differences in DNA methylation between the two Romanian adoption groups of same ethnicity. No further covariates were included in this test, as all samples were taken at the same age. The potential confounding effect of sex on the identified differences was ruled out through the comparison of results with a sex-regressed model (Supplementary Figure 1). Associations between DNA methylation and exposure time (continuous) as well as IQ and ToM were analyzed using linear regression. Region-level analysis for deprivation group, ToM and IQ was performed by spatially combining correlated *P*-values using the Python module *comb-p.*^30^ We allowed a maximum distance of 1000 bp between neighboring CpG sites, and only included probes with a *P*-value<0.05 in the initial epigenome wide association scan as starting points for identifying potential differentially methylated regions (DMRs). For each DMR, we report the combined *P*, which is Stouffer–Liptak–Kechris-corrected for regional correlation structure, and the multiple-testing-corrected Šidák *P*-value. The latter corrects the combined *P* for *n*_a_/*n*_r_ tests, where *n*_a_ is the total number of probes tested in the initial epigenome wide association scan and *n*_r_ the number of probes in the given region. The Bioconductor package *bumphunter*^31^ was used to confirm DMRs identified by *comb-p* with an alternative method. We report the empirical *P*-value, calculated using 1000 permutations. Genes were assigned to probes using the Genomic Regions Enrichment of Annotations Tool (GREAT) package from the Bejerano Lab at Stanford University (http://bejerano.stanford.edu/great/public/html),^32^ taking into account the functional significance of *cis*-regulatory regions.

### Bisulfite-pyrosequencing

To technically validate the 450K array data at the *CYP2E1* DMR, a bisulfite-pyrosequencing assay spanning three CpG sites (cg14250048, cg00436603 and cg01465364) was designed using the PyroMark Assay design software (Qiagen, Hilden, Germany). Bisulfite-PCR amplification was performed in duplicate on samples with sufficient remaining DNA using the primers in Supplementary Table 3 and a PCR annealing temperature of 55 °C. DNA methylation was quantified in a subset of 36 samples with sufficient remaining DNA using the Pyromark Q24 system (Qiagen), following the manufacturer's standard instructions, and the Pyro Q24 CpG 2.0.6 software.

## Results

### Sociocognitive consequences of exposure

**Figure 1:**
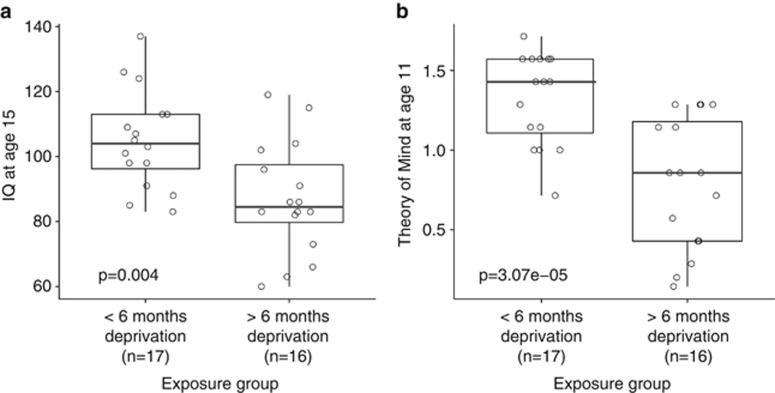
Exposure to severe early-life deprivation is negatively associated with performance in sociocognitive tasks in the English and Romanian Adoptees study (ERA) subsample included in methylomic profiling. Prolonged exposure (⩾6 months) was significantly associated with lower scores for (**a**) intelligence quotient (IQ) at the age of 15 (*P*=0.004) (**b**) and the Theory of Mind at the age of 11 (*P*=3.07 × 10^−4^).

Phenotypic analyses on the selected subsample of the ERA cohort used in this study confirmed previously reported negative associations between exposure to severe early-life institutional deprivation and performance in sociocognitive tests (Figure 1). Romanian adoptees exposed to >6 months of deprivation scored significantly lower on tests of both IQ (*P*=0.004) and ToM (*P*=3.07 × 10^−4^).

### Deprivation-associated DNA methylation differences

We first assessed DNA methylation differences at specific 450K array probes between Romanian adoptees categorized as having experienced ‘limited' (<6 months in institutional deprivation, *n*=17) and ‘extended' periods of institutional deprivation (>6 months in institutional deprivation, *n*=16; see Table 1 and Supplementary Figure 2 for the top-ranked differentially methylated positions). No probe-wise differences remained significant after correction for multiple correction, although this is not surprising, given the small number of samples available for this study. DNA methylation differences for the 100 top-ranked exposure-associated differentially methylated positions (Supplementary Table 4) were highly correlated with effect sizes at the same loci in a quantitative analysis of exposure duration (*r*=0.93, *P*=3.03 × 10^−44^; Supplementary Figure 3), indicating that the effects of severe deprivation at these loci are likely to be cumulative.

**Figure 2:**
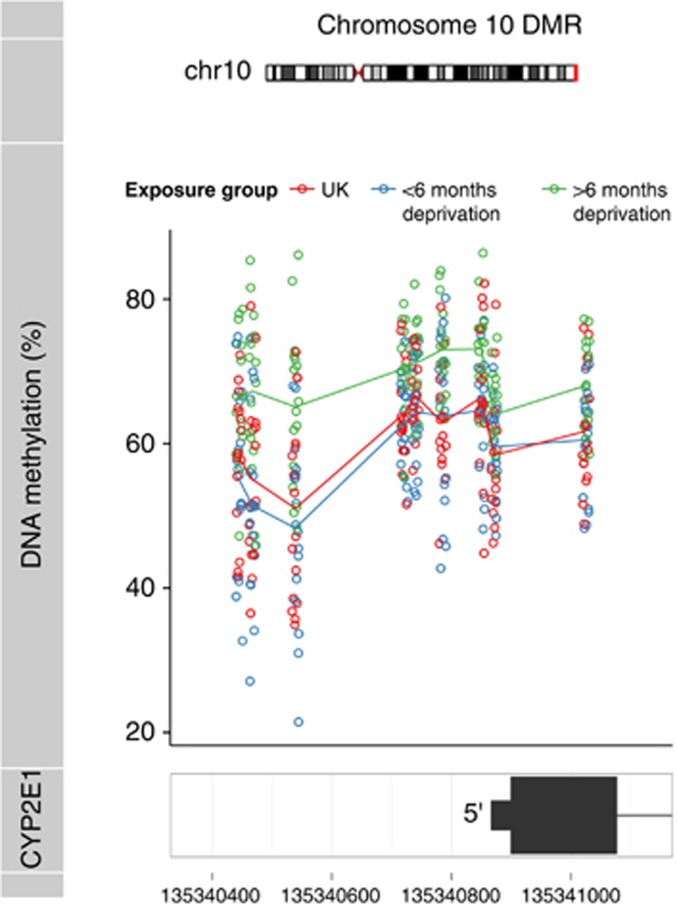
A differentially methylated region (DMR) spanning the transcription start site of *CYP2E1* shows significantly increased DNA methylation levels in adoptees exposed to severe early-life adversity. A DMR on chromosome 10 spanning nine sequential 450K array probes (chr10:135340445-135341026) was identified by *comb-p.* DNA methylation across this region is significantly elevated (combined *P*=2.21 × 10^−10^; corrected Šidák *P*=2.98 × 10^−5^) in individuals exposed to severe long-term (⩾6 months) institutional deprivation compared with the low-exposure (<6 months) group and UK control group. Data for an extended region around this DMR are shown in Supplementary Figure 4.

We next used *comb-p*^30^ to identify spatially correlated regions of differential DNA methylation, identifying a significant DMR on chromosome 10 spanning nine sequential 450K array probes, which were consistently increased in DNA methylation in the severe early institutional deprivation group (combined *P*=2.21 × 10^−10^, corrected Šidák *P*=2.98 × 10^−5^). This region was also identified using an alternative DMR analysis method (*bumphunter*^31^) as showing significantly elevated DNA methylation in the severely exposed group (adjusted *P*=0.002; Figure 2, Supplementary Figure 4 and Table 2). By comparing the two Romanian adoptee groups with the matched group of children born and adopted within the UK, we were able to show that increased DNA methylation across this DMR is specific to the group that experienced extended deprivation; the control group of UK adoptees was indistinguishable from the Romanian group adopted before the age of 6 months at each of the nine CpG sites comprising the DMR (Figure 2 and Supplementary Figure 4). This ~600bp DMR spans the transcription start site and first exon of the cytochrome P450 gene, *CYP2E1.* There was a significant correlation between DNA methylation levels independently derived from the 450K array and bisulfite-pyrosequencing experiments (*r*=0.52, *P*=0.001, Supplementary Figure 5).

### Association between DNA methylation and deprivation-related sociocognitive and intellectual impairments

**Figure 3:**
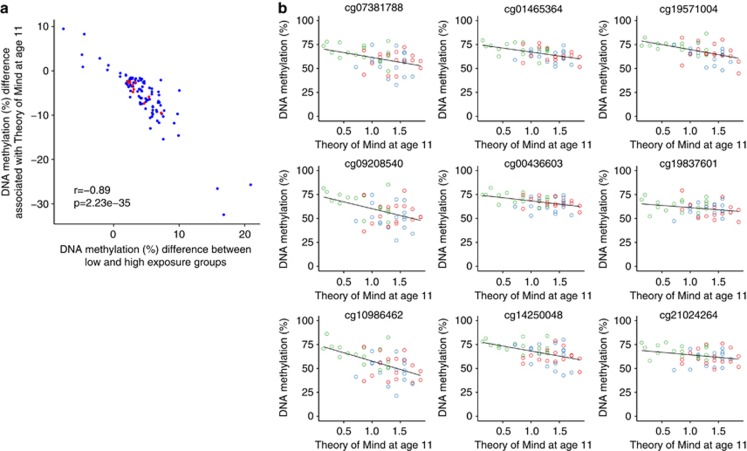
Exposure-associated DNA methylation variation is associated with Theory of Mind test performance. (**a**) For the 100 top-ranked exposure-associated differentially methylated positions (DMPs; see Supplementary Table 4), effect sizes for association with exposure group correlated significantly with effect sizes for association with Theory of Mind at the age of 11 (*r*=−0.89, *P*=2.23 × 10^−35^). The top 10 exposure-associated DMPs (see Table 1) are highlighted in red. (**b**) The *CYP2E1* DMR associated with exposure group is also significantly associated with the Theory of Mind (combined *P*=4.87 × 10^−9^, corrected Šidák *P*=8.79 × 10^−5^). Associations between DNA methylation and Theory of Mind are shown for the nine probes constituting the DMR, with the previously used coloring scheme (UK adoptees: red, short deprivation exposure group: blue, extended exposure group: green).

We next tested whether exposure-associated DNA methylation differences were associated with established deprivation-related impairments in cognition and deficits in ToM across samples for which 450K array data were available. For the 100 top-ranked exposure-group differentially methylated positions, there was a highly significant negative correlation between exposure-group DNA methylation differences and effect sizes at the same probes for both IQ (*r*=−0.82, *P*=4.48 × 10^−25^, Supplementary Figure 6) and ToM (*r*=−0.89, *P*=2.23 × 10^−35^, Figure 3a). Furthermore, using *comb-p* to identify DMRs for sociocognitive and intellectual impairments, we found that DNA methylation across the nine CpG sites in the deprivation-associated *CYP2E1* DMR on chromosome 10 was significantly associated with ToM (combined *P*=4.87 × 10^−9^; Table 3 and Figure 3b) and cognitive impairment (combined *P*=2.912 × 10^−5^).

## Discussion

Using samples from a unique ‘natural experiment' following children exposed to prolonged severe institutional deprivation, we provide evidence for significant alterations in DNA methylation in response to severe early-life social adversity in humans. We identified a DMR that was associated with extended institutional deprivation across nine adjacent CpG sites spanning the transcription start site and first exon of the cytochrome P450 gene *CYP2E1.* Elevated DNA methylation across this DMR was specific to the group exposed to more than 6 months in Romanian institutions; early-adopted Romanian adoptees were indistinguishable from the control group of UK adoptees—an effect that mirrors prior findings relating deprivation and psychiatric disorders and cognition.^13^ DNA methylation across the nine CpG sites in the *CYP2E1* DMR was also associated with ToM performance and cognitive impairment.

The CYP2E1 protein is a member of the cytochrome P450 (CYPs) super family of enzymes, with a role in the metabolism of various exogenous compounds including drugs of abuse and neurotoxins.^33^ It is also involved in gluconeogenesis and the synthesis of cholesterol, steroids and other lipids.^34^ *CYP2E1* is most abundantly expressed in the liver, although like other CYPs it is present and is active in the human brain, including the frontal cortex, hippocampus, amygdala, hypothalamus and cerebellum (GTEx Analysis Release V4: dbGaP Accession phs000424.v4.p1 (ref. 33)). There is evidence to suggest that CYPs in the brain may have a role in modulating behavior^35^ and cognitive processes (for example, shown by imaging genetic studies^36^) as well as susceptibility to central nervous system diseases and drug dependence.^37^

It is currently unknown which molecular pathways in the brain might be affected by changes in *CYP2E1* function, and how deprivation-related sociocognitive deficits might be mechanistically connected to epigenetic variation regulating *CYP2E1*. Of note, increased methylation of a CpG site in close proximity (<1 kb) to our DMR in neonates has been recently associated with prenatal exposure to selective serotonin reuptake inhibitors^38^ (Supplementary Figure 4). Although this specific CpG site was not within the DMR identified in our study, it was nominally significantly associated with exposure to adversity (*P*=0.034). Prenatal selective serotonin reuptake inhibitor exposure, similar to severe early-life adversity, has been implicated as a risk factor for long-term cognitive deficits and psychopathology.^39^ In a mouse model, it was shown that chronic psychoemotional stress reduced CYP2E1 protein levels by one half, suggesting that stress exposure might have a role in CYP2E1 regulation.^40^ Our observation that DNA methylation differences in the regulatory region of *CYP2E1* associated with extended deprivation also correlated with reduced IQ, and ToM is consistent with the view that shared processes may be involved in mediating the observed deficits in both social and intellectual functioning.^17, 41^

There is now a large body of evidence and a strong, scientific consensus that childhood stress and early adversity, especially in such extreme forms as the institutional deprivation experienced by the ERA sample, are associated with disturbances of childhood mental health and life-long risks of chronic disorders of mental and physical health.^42^ The exact mechanisms by which signals from the social environment impinge on the developing brain to shape the neural circuitry and what role epigenetic processes may have in stabilizing developmental trajectories across the lifespan are only just beginning to be elucidated. Brain structure and function are especially responsive to experience early in life, and development is characterized by the key developmental stages of heightened plasticity.^43^ Recent research shows that during these critical periods the genome may be particularly vulnerable to epigenetic disruption.^44^ With regard to the ERA sample, it can be speculated that the lack of emotional, sensory and cognitive stimulation associated with deprivation of personalized care during sensitive periods in infant life might have led to epigenetic changes resulting in insufficient fine-tuning of the brain circuitry mediating socioemotional behaviors and underlying higher cognitive function.

One previous study has investigated epigenetic alterations in 8-year-old institutionalized children. Differential methylation patterns (at an extremely non-stringent uncorrected *P*<0.01) were found at 914 CpG sites, with ~90% of these nominally differentially methylated sites showing elevated methylation in the institutionalized group.^45^ In addition to the analytical differences between the studies, samples in this prior study were not exposed to significant deprivation, which may explain the difference in number and magnitude of exposure-associated changes.

Our study has a number of important limitations. First, the number of samples profiled in this study is small, and replication in cohorts with similar types of deprivation experience is warranted. However, the ERA represents a unique natural experiment cohort, and access to equivalent samples exposed to a similar level of adversity for replication is by necessity difficult. Second, the small number of samples means that it is underpowered to formally assess whether deprivation effects on sociocognitive processes are mediated by epigenetic effects. Furthermore, our analyses were cross-sectional, and it is not possible to causally link exposure to the variation we observe. It cannot be ruled out that the observed differences in DNA methylation were caused by deprivation-related impairments observed in the high-risk group, or by other confounding factors such as adverse prenatal conditions (although no differences in birth weight were observed between the long- and short-exposed Romanian adoptees) or medication use. Finally, the observed differences in DNA methylation were observed in buccal cells, and the extent to which peripheral markers index epigenetic variation in central nervous tissue is still debated.^12, 46^ Of note, buccal cells derive from the same embryonic cells as brain tissue (ectoderm) and have less cellular heterogeneity compared with whole blood.^47^ Although there are well-documented tissue-specific differences in DNA methylation, exposure-associated changes in DNA methylation can be identified in many cell types, and peripheral tissues may have some utilities as potential biomarkers of exposure or disease.^12, 46, 48^ Despite these limitations, our data are consistent with the notion that environmental insults of sufficient biological impact during early development might be associated with epigenetic variation detectable in peripheral cells, and provide further support for a role of epigenetic processes in linking the effects of early-life adversity to cognitive and neurobiological phenotypes.

To conclude, children exposed to extreme early institutional deprivation were characterized by significantly increased DNA methylation across a region of the *CYP2E1* gene with putative functional significance for brain function. These findings support the notion that severe social adversity may induce epigenetic variation in human subjects. Future studies should replicate and extend this finding with larger samples and investigate longitudinal changes in DNA methylation over time as a function of post-adversity environments and genomic variations, and relate these to changes in phenotype. It will be important to further investigate the neurobiological significance of these changes by linking DMRs to brain structure and function.

## Figures and Tables

**Table 1:** The top-ranked differentially methylated positions associated with exposure to severe institutional deprivation

*Probe*	*GREAT gene annotation*	P-*value*	*DNA methylation difference*	*>6-Month exposure (mean)*	*<6-Month exposure (mean)*
cg11634248	CHKA, SUV420H1	2.35 × 10^−^^5^	0.03	0.88	0.85
cg14272935	FGF5	6.89 × 10^−5^	0.05	0.37	0.32
cg16668903	SNX24, PPIC	9.75 × 10^−5^	0.07	0.79	0.72
cg06969206	HHIPL1	1.08 × 10^−4^	0.05	0.24	0.19
cg22982014	LGALS4, HNRNPL	1.26 × 10^−4^	0.03	0.24	0.21
cg24843511	S100A2, S100A16	1.38 × 10^−4^	0.03	0.82	0.79
cg18015809	GPR110, TNFRSF21	1.60 × 10^−4^	0.03	0.90	0.88
cg08157194	SLC25A17, MCHR1	1.63 × 10^−4^	0.02	0.89	0.87
cg04213775	SLC12A7, NKD2	1.68 × 10^−4^	0.03	0.85	0.82
cg07085824	SGK196	1.72 × 10^−4^	0.02	0.13	0.10

Abbreviation: GREAT, Genomic Regions Enrichment of Annotations Tool.

**Table 2:** Association statistics for the nine individual probes in the chromosome 10 DMR

*Probe*	*Position*	P *>6-month- versus <6-month exposure*	P *UK versus <6-month exposure*	*DNA methylation difference >6-month- versus <6-month exposure*	*>6-Month exposure (mean)*	*<6-Month exposure (mean)*	*UK (mean)*
cg07381788	135 340 445	0.003	0.419	0.12	0.67	0.55	0.58
cg09208540	135 340 467	7.71 × 10^−4^	0.408	0.16	0.67	0.51	0.55
cg10986462	135 340 539	3.00 × 10^−4^	0.545	0.17	0.65	0.48	0.51
cg01465364	135 340 721	0.001	0.554	0.08	0.70	0.63	0.64
cg00436603	135 340 740	0.007	0.316	0.07	0.71	0.64	0.67
cg14250048	135 340 785	0.010	0.790	0.09	0.73	0.64	0.63
cg19571004	135 340 850	0.003	0.586	0.08	0.73	0.65	0.66
cg19837601	135 340 871	0.053	0.666	0.04	0.64	0.60	0.58
cg21024264	135 341 025	0.004	0.660	0.08	0.68	0.61	0.62

Abbreviation: DMR, differentially methylated region.

**Table 3:** Association statistics of the nine probes in the chromosome 10 DMR with Theory of Mind

*Probe*	*Position*	P*-value*	*Effect size*^a^
cg07381788	135 340 445	0.006	−0.19
cg09208540	135 340 467	0.001	−0.27
cg10986462	135 340 539	1.10 × 10^−4^	−0.32
cg01465364	135 340 721	2.78 × 10^−4^	−0.15
cg00436603	135 340 740	0.002	−0.13
cg14250048	135 340 785	0.001	−0.20
cg19571004	135 340 850	3.12 × 10^−4^	−0.20
cg19837601	135 340 871	0.072	−0.08
cg21024264	135 341 025	0.052	−0.10

a Abbreviations: DMR, differentially methylated region. Effect size is scaled to the maximum Theory of Mind score.

## Supplementary Material

Supplementary Informations
