# Supplementary material for: Severe psychosocial deprivation in early childhood is associated with increased DNA methylation across a region spanning the transcription start site of CYP2E1
Source: Transl Psychiatry. 2016 Jun 7;6(6):e830–. doi: 10.1038/tp.2016.95 (PMC4931613; doi:10.1038/tp.2016.95)
Supplement: Supplementary Informations [file tp201695x1.doc]

**SUPPLEMENTARY MATERIAL**

| **Supplementary Table 1. Demographic information on samples. Associations between exposure and cognitive and social cognitive outcome measures are reported.** | | | | |
| --- | --- | --- | --- | --- |
|  | **>6 months deprivation (n=16)** | **<6 months deprivation (n=17)** | **UK (n=16)** | ***P* >6 months vs <6 months derivation^*^** |
| Sex (m/f) | 7/9 | 9/8 | 10/6 | 0.732 |
| Mean deprivation time (months) | 20.69 | 3.47 | - | 1.41 × 10^-5^ |
| Mean IQ at age 15 | 87.00 | 105.06 | 105.75 | 0.004 |
| Mean Theory of Mind at age 11 | 0.80 | 1.32 | 1.38 | 3.07 × 10^-4^ |
| * *P* values were calculated using a Fisher’s exact test for sex and a two-sample t-test for deprivation time, IQ and Theory of Mind | | | | |

| **Supplementary Table 2. Sample characteristics for potential confounders – including birth weight and substance use.** | | | | |
| --- | --- | --- | --- | --- |
|  | **>6 months deprivation (n=16)** | **<6 months deprivation (n=17)** | **UK (n=16)** | ***P* >6 months vs <6 months derivation^*^** |
| **Birth weight (kg)** | 2.86 | 2.96 | 3.28 | 0.557 |
| **Smoking, alcohol, drug abuse (yes/no)** | | | | |
| Self-report |  |  |  |  |
| Smoking (>5 cigarettes/day) | 1/15 | 2/16 | 5/16 | 0.525 |
| Any sign of alcohol abuse | 3/16 | 3/16 | 4/15 | 0.641 |
| Occasional cannabis use | 1/15 | 2/15 | 3/15 | 0.500 |
| Hard Drug Use | 0/15 | 0/16 | 2/15 | - |
| Parent report |  |  |  |  |
| Smoking (>5 cigarettes/day) | 0/15 | 0/17 | 0/16 | - |
| Any sign of alcohol abuse | 1/16 | 3/17 | 2/16 | 0.324 |
| Occasional cannabis use | 1/16 | 0/16 | 0/16 | 0.500 |
| Drug use | 0/15 | 0/17 | 0/16 | - |
| * *P* values were calculated using a Fisher’s exact test for smoking, alcohol, cannabis and other drug use and a two-sample t-test for birth weight. | | | | |

| **Supplementary Table 3. Primers used for pyrosequencing validation.** | | |
| --- | --- | --- |
| **Sequencing Primer** | **Forward Primer** | **Reverse Primer** |
| TTTTTTAGAATATATTATAAAATT | 5’-GGTATTGGTTGGTGGGTTATT-3’ | 5’-Biosg-TACTATACACCTACCTCCACATAAACAC-3’ |

| **Supplementary Table 4. Top 100 differentially methylated probes associated with exposure (>6 months vs <6 months exposure). Association between methylation at the top 100 DMPs and deprivation time (quantitative), IQ and Theory of Mind are also reported.** | | | | | | | | | | | |
| --- | --- | --- | --- | --- | --- | --- | --- | --- | --- | --- | --- |
|  |  |  | **GREAT_gene annotation** | **Exposure group** | | **Deprivation time** | | **IQ** | | **Theory of Mind** | |
| **Rank** | **Probe** | **Position** |  | ***P* value** | **Effect** | ***P* value** | **Effect** | ***P* value** | **Effect** | ***P* value** | **Effect** |
| 1 | cg11634248 | 11 : 67926133 | CHKA, SUV420H1 | 2.35 × 10 ^-5^ | 0.03 | 0.004 | 0.04 | 0.052 | -0.06 | 0.113 | -0.03 |
| 2 | cg14272935 | 4 : 81185765 | FGF5 | 6.89 × 10 ^-5^ | 0.05 | 0.067 | 0.04 | 0.250 | -0.06 | 0.021 | -0.06 |
| 3 | cg16668903 | 5 : 122206322 | SNX24, PPIC | 9.75× 10 ^-5^ | 0.07 | 0.109 | 0.05 | 0.049 | -0.13 | 0.007 | -0.09 |
| 4 | cg06969206 | 14 : 100111183 | HHIPL1 | 1.08 × 10 ^-4^ | 0.05 | 0.005 | 0.06 | 0.037 | -0.09 | 0.002 | -0.07 |
| 5 | cg22982014 | 19 : 39322497 | LGALS4, HNRNPL | 1.26 × 10 ^-4^ | 0.03 | 0.031 | 0.03 | 0.020 | -0.07 | 0.002 | -0.05 |
| 6 | cg24843511 | 1 : 153579799 | S100A2, S100A16 | 1.38 × 10 ^-4^ | 0.03 | 0.009 | 0.03 | 0.255 | -0.03 | 0.022 | -0.03 |
| 7 | cg18015809 | 6 : 47202254 | GPR110, TNFRSF21 | 1.60 × 10 ^-4^ | 0.03 | 0.070 | 0.02 | 0.021 | -0.06 | 0.068 | -0.03 |
| 8 | cg08157194 | 22 : 41185264 | SLC25A17, MCHR1 | 1.63 × 10 ^-4^ | 0.02 | 5.53 × 10 ^-4^ | 0.03 | 0.006 | -0.06 | 0.023 | -0.03 |
| 9 | cg04213775 | 5 : 1063087 | SLC12A7, NKD2 | 1.68 × 10 ^-4^ | 0.03 | 0.025 | 0.03 | 0.028 | -0.07 | 0.019 | -0.04 |
| 10 | cg07085824 | 8 : 42948105 | SGK196 | 1.72 × 10 ^-4^ | 0.02 | 0.062 | 0.02 | 0.136 | -0.04 | 0.102 | -0.02 |
| 11 | cg02132051 | 14 : 73020007 | DPF3, RGS6 | 1.84 × 10 ^-4^ | 0.04 | 0.078 | 0.03 | 0.174 | -0.06 | 0.013 | -0.06 |
| 12 | cg24780167 | 2 : 179744587 | TTN, CCDC141 | 1.98 × 10 ^-4^ | 0.06 | 0.041 | 0.05 | 0.270 | -0.06 | 0.009 | -0.08 |
| 13 | cg11708358 | 6 : 150183516 | LRP11, PCMT1 | 2.22 × 10 ^-4^ | 0.09 | 0.023 | 0.09 | 0.003 | -0.25 | 0.011 | -0.12 |
| 14 | cg14441262 | 6 : 33140769 | COL11A2, HLA-DPB1 | 2.22 × 10 ^-4^ | 0.05 | 0.034 | 0.04 | 0.300 | -0.05 | 0.291 | -0.03 |
| 15 | cg08241115 | 16 : 722688 | RHBDL1 | 2.26 × 10 ^-4^ | 0.03 | 0.002 | 0.04 | 0.146 | -0.05 | 0.059 | -0.03 |
| 16 | cg21483883 | 7 : 37487352 | AOAH, ELMO1 | 2.27 × 10 ^-4^ | -0.05 | 0.152 | -0.03 | 0.279 | 0.05 | 0.165 | 0.04 |
| 17 | cg16699861 | 16 : 30429204 | ZNF771, DCTPP1 | 2.45 × 10 ^-4^ | 0.01 | 0.003 | 0.01 | 0.344 | -0.01 | 0.321 | -0.01 |
| 18 | cg21145140 | 5 : 110409467 | WDR36, TSLP | 2.69 × 10 ^-4^ | 0.07 | 0.004 | 0.08 | 0.020 | -0.14 | 0.004 | -0.09 |
| 19 | cg25021532 | 8 : 107782513 | ABRA | 2.86 × 10 ^-4^ | 0.04 | 0.423 | 0.02 | 0.834 | -0.01 | 0.428 | -0.02 |
| 20 | cg14566624 | 1 : 6321582 | GPR153 | 2.91 × 10 ^-4^ | 0.07 | 0.005 | 0.09 | 0.078 | -0.11 | 0.032 | -0.07 |
| 21 | cg10986462 | 10 : 135340539 | CYP2E1 | 3.00 × 10 ^-4^ | 0.17 | 0.014 | 0.19 | 7.57 × 10 ^-4^ | -0.53 | 1.10 × 10 ^-4^ | -0.32 |
| 22 | cg13628514 | 12 : 110271439 | TRPV4 | 3.00 × 10 ^-4^ | 0.06 | 0.037 | 0.06 | 0.810 | -0.01 | 0.105 | -0.05 |
| 23 | cg18167179 | 14 : 74058881 | ACOT4 | 3.01 × 10 ^-4^ | -0.01 | 0.236 | -0.01 | 0.426 | 0.01 | 0.697 | 0.00 |
| 24 | cg13443165 | 9 : 33130375 | SMU1, B4GALT1 | 3.13 × 10 ^-4^ | 0.21 | 0.002 | 0.28 | 0.062 | -0.39 | 0.022 | -0.26 |
| 25 | cg26385743 | 2 : 131101463 | CCDC115, IMP4 | 3.18 × 10 ^-4^ | 0.05 | 0.088 | 0.04 | 0.153 | -0.08 | 0.027 | -0.07 |
| 26 | cg08397920 | 16 : 2258580 | C16orf79, MLST8 | 3.56 × 10 ^-4^ | 0.03 | 0.038 | 0.03 | 0.092 | -0.05 | 0.351 | -0.02 |
| 27 | cg07805911 | 7 : 818287 | SUN1, PRKAR1B | 3.66 × 10 ^-4^ | 0.05 | 0.008 | 0.07 | 0.670 | -0.02 | 0.019 | -0.06 |
| 28 | cg22443762 | 1 : 197890608 | NEK7, LHX9 | 3.82 × 10 ^-4^ | 0.07 | 0.053 | 0.06 | 0.219 | -0.08 | 0.035 | -0.07 |
| 29 | cg03131097 | 10 : 43802881 | FXYD4, RASGEF1A | 4.00 × 10 ^-4^ | 0.03 | 0.012 | 0.03 | 0.001 | -0.09 | 0.020 | -0.04 |
| 30 | cg20011352 | 8 : 37655269 | GPR124 | 4.23 × 10 ^-4^ | 0.02 | 0.015 | 0.03 | 0.148 | -0.03 | 0.001 | -0.04 |
| 31 | cg12748499 | 1 : 244374172 | ZNF238, ADSS | 4.32 × 10 ^-4^ | 0.04 | 0.013 | 0.04 | 0.271 | -0.04 | 0.049 | -0.04 |
| 32 | cg09206774 | 10 : 3581233 | PITRM1, KLF6 | 4.38 × 10 ^-4^ | 0.05 | 0.021 | 0.05 | 0.423 | -0.04 | 0.358 | -0.02 |
| 33 | cg16268769 | 1 : 156890782 | PEAR1, ARHGEF11 | 4.61 × 10 ^-4^ | 0.04 | 0.017 | 0.05 | 0.041 | -0.10 | 0.002 | -0.08 |
| 34 | cg06705986 | 10 : 86004888 | LRIT1, RGR | 4.88 × 10 ^-4^ | 0.08 | 0.213 | 0.05 | 0.011 | -0.18 | 0.006 | -0.10 |
| 35 | cg18170229 | 12 : 322587 | SLC6A12 | 5.08 × 10 ^-4^ | 0.06 | 0.012 | 0.07 | 0.039 | -0.11 | 0.004 | -0.09 |
| 36 | cg13898430 | 1 : 25292274 | RUNX3, SYF2 | 5.10 × 10 ^-4^ | 0.04 | 0.008 | 0.05 | 0.016 | -0.10 | 0.050 | -0.04 |
| 37 | cg12973753 | 6 : 26208128 | HIST1H4E, HIST1H2BG | 5.17 × 10 ^-4^ | 0.03 | 0.019 | 0.03 | 0.084 | -0.05 | 0.257 | -0.02 |
| 38 | cg08064292 | 15 : 98196234 | ARRDC4 | 5.52 × 10 ^-4^ | 0.10 | 0.122 | 0.07 | 0.130 | -0.15 | 0.067 | -0.10 |
| 39 | cg09000469 | 10 : 134229757 | INPP5A, STK32C | 5.53 × 10 ^-4^ | 0.04 | 0.035 | 0.04 | 0.139 | -0.06 | 0.326 | -0.02 |
| 40 | cg18243598 | 7 : 2172011 | MAD1L1, ELFN1 | 5.67 × 10 ^-4^ | 0.02 | 0.092 | 0.02 | 0.049 | -0.05 | 0.180 | -0.02 |
| 41 | cg12637397 | 5 : 1229127 | SLC6A18, TERT | 5.72 × 10 ^-4^ | 0.03 | 0.158 | 0.02 | 0.049 | -0.06 | 0.236 | -0.02 |
| 42 | cg19099736 | 22 : 29702810 | GAS2L1 | 5.86 × 10 ^-4^ | 0.03 | 0.005 | 0.04 | 0.398 | -0.03 | 0.338 | -0.02 |
| 43 | cg20203971 | 2 : 240171099 | HDAC4, TWIST2 | 5.97 × 10 ^-4^ | 0.02 | 0.004 | 0.03 | 0.005 | -0.06 | 0.009 | -0.03 |
| 44 | cg07335357 | 17 : 77758850 | CBX2, CBX8 | 6.14 × 10 ^-4^ | 0.02 | 0.030 | 0.02 | 0.661 | 0.01 | 0.341 | -0.01 |
| 45 | cg05914582 | 12 : 6588003 | VAMP1, MRPL51 | 6.18 × 10 ^-4^ | 0.03 | 0.012 | 0.03 | 0.003 | -0.08 | 0.002 | -0.05 |
| 46 | cg04522915 | 20 : 646942 | SRXN1, SCRT2 | 6.59 × 10 ^-4^ | 0.04 | 0.036 | 0.04 | 0.020 | -0.11 | 0.020 | -0.06 |
| 47 | cg01164118 | 3 : 74571289 | CNTN3 | 6.87 × 10 ^-4^ | -0.05 | 0.076 | -0.04 | 0.368 | 0.04 | 0.730 | 0.01 |
| 48 | cg15889768 | 17 : 42619247 | GPATCH8, FZD2 | 6.92 × 10 ^-4^ | 0.04 | 0.076 | 0.03 | 0.260 | -0.04 | 0.020 | -0.05 |
| 49 | cg13392468 | 9 : 139701199 | C9orf86 | 6.95 × 10 ^-4^ | 0.06 | 0.162 | 0.04 | 0.908 | -0.01 | 0.140 | -0.05 |
| 50 | cg01573067 | 6 : 168067266 | TCP10, MLLT4 | 7.55 × 10 ^-4^ | 0.06 | 0.004 | 0.08 | 0.010 | -0.14 | 7.34 × 10 ^-4^ | -0.09 |
| 51 | cg09208540 | 10 : 135340467 | CYP2E1 | 7.71 × 10 ^-4^ | 0.16 | 0.061 | 0.15 | 0.004 | -0.44 | 0.001 | -0.27 |
| 52 | cg21648202 | 19 : 14171331 | PALM3 | 7.89 × 10 ^-4^ | 0.07 | 0.115 | 0.05 | 0.173 | -0.10 | 0.060 | -0.08 |
| 53 | cg08144503 | 16 : 67450471 | ZDHHC1 | 7.91 × 10 ^-4^ | 0.03 | 0.010 | 0.04 | 0.048 | -0.07 | 0.197 | -0.03 |
| 54 | cg23709618 | 4 : 119273073 | PRSS12 | 8.19 × 10 ^-4^ | 0.04 | 0.021 | 0.05 | 0.003 | -0.12 | 0.005 | -0.06 |
| 55 | cg07509161 | 14 : 54552632 | CDKN3, BMP4 | 8.52 × 10 ^-4^ | -0.04 | 0.023 | -0.05 | 0.228 | 0.05 | 4.08 × 10 ^-4^ | 0.08 |
| 56 | cg00368296 | 19 : 8052183 | TIMM44, ELAVL1 | 8.66 × 10 ^-4^ | 0.04 | 0.132 | 0.03 | 0.651 | -0.02 | 0.279 | -0.02 |
| 57 | cg05387404 | 3 : 47888039 | DHX30, MAP4 | 8.73 × 10 ^-4^ | 0.03 | 0.040 | 0.03 | 0.395 | -0.02 | 0.038 | -0.03 |
| 58 | cg16005818 | 1 : 7700504 | VAMP3, CAMTA1 | 8.78 × 10 ^-4^ | -0.01 | 6.12 × 10 ^-4^ | -0.01 | 2.77 × 10 ^-4^ | 0.03 | 0.001 | 0.01 |
| 59 | cg26380116 | 12 : 125927452 | TMEM132B | 8.86 × 10 ^-4^ | 0.04 | 0.011 | 0.05 | 0.452 | -0.04 | 0.163 | -0.04 |
| 60 | cg17313945 | 6 : 32977983 | HLA-DOA | 9.10 × 10 ^-4^ | 0.05 | 0.397 | 0.02 | 0.274 | -0.06 | 0.037 | -0.06 |
| 61 | cg00476944 | 7 : 157411142 | DNAJB6, PTPRN2 | 9.35 × 10 ^-4^ | 0.04 | 0.038 | 0.04 | 0.001 | -0.15 | 0.009 | -0.06 |
| 62 | cg00958409 | 1 : 155023062 | ADAM15 | 9.39 × 10 ^-4^ | 0.03 | 0.010 | 0.03 | 0.138 | -0.07 | 0.018 | -0.06 |
| 63 | cg16008138 | 17 : 60885892 | MARCH10 | 9.88 × 10 ^-4^ | 0.04 | 0.083 | 0.04 | 0.131 | -0.07 | 0.002 | -0.07 |
| 64 | cg23053407 | 1 : 104068312 | RNPC3 | 9.89 × 10 ^-4^ | 0.04 | 0.042 | 0.04 | 0.638 | -0.03 | 0.128 | -0.05 |
| 65 | cg00171161 | 4 : 88343636 | NUDT9 | 0.001 | 0.01 | 0.010 | 0.01 | 0.076 | -0.02 | 0.072 | -0.01 |
| 66 | cg04636557 | 1 : 231472424 | C1orf124, EXOC8 | 0.001 | 0.07 | 0.049 | 0.07 | 0.404 | -0.07 | 0.009 | -0.12 |
| 67 | cg00053916 | 8 : 37457329 | ZNF703, KCNU1 | 0.001 | 0.06 | 0.016 | 0.07 | 0.051 | -0.13 | 0.014 | -0.08 |
| 68 | cg19506311 | 7 : 994742 | ADAP1 | 0.001 | 0.07 | 0.032 | 0.08 | 0.022 | -0.19 | 0.032 | -0.10 |
| 69 | cg02334521 | 10 : 324041 | ZMYND11, DIP2C | 0.001 | 0.02 | 0.015 | 0.02 | 0.003 | -0.06 | 0.031 | -0.02 |
| 70 | cg04770705 | 16 : 28509428 | APOBR, IL27 | 0.001 | 0.05 | 0.270 | 0.03 | 0.080 | -0.10 | 0.201 | -0.04 |
| 71 | cg03272089 | 19 : 2191261 | SF3A2, DOT1L | 0.001 | 0.02 | 0.003 | 0.03 | 0.001 | -0.09 | 0.194 | -0.02 |
| 72 | cg19786808 | 17 : 71431877 | CDC42EP4, SDK2 | 0.001 | 0.04 | 0.002 | 0.06 | 0.033 | -0.09 | 8.51 × 10 ^-4^ | -0.07 |
| 73 | cg18101118 | 3 : 45017337 | EXOSC7, ZDHHC3 | 0.001 | 0.07 | 0.168 | 0.05 | 0.069 | -0.13 | 0.161 | -0.05 |
| 74 | cg14375923 | 13 : 91827042 | GPC5 | 0.001 | 0.05 | 0.011 | 0.06 | 0.173 | -0.08 | 0.005 | -0.09 |
| 75 | cg06801943 | 1 : 2101479 | SKI, PRKCZ | 0.001 | 0.05 | 0.047 | 0.05 | 0.086 | -0.09 | 0.016 | -0.07 |
| 76 | cg01465364 | 10 : 135340721 | CYP2E1 | 0.001 | 0.08 | 0.007 | 0.10 | 0.003 | -0.24 | 2.78 × 10 ^-4^ | -0.15 |
| 77 | cg20610031 | 4 : 1950081 | WHSC2, WHSC1 | 0.001 | 0.03 | 0.002 | 0.04 | 0.024 | -0.07 | 0.108 | -0.03 |
| 78 | cg16498741 | 8 : 41144520 | ZMAT4, SFRP1 | 0.001 | 0.10 | 0.039 | 0.10 | 0.150 | -0.16 | 0.012 | -0.15 |
| 79 | cg10013969 | 7 : 128003601 | PRRT4 | 0.001 | -0.08 | 0.026 | -0.09 | 0.021 | 0.22 | 0.075 | 0.09 |
| 80 | cg03440989 | 17 : 40155391 | DNAJC7, CNP | 0.001 | 0.03 | 0.066 | 0.03 | 0.199 | -0.03 | 0.012 | -0.03 |
| 81 | cg02420027 | 13 : 41632640 | WBP4 | 0.001 | 0.04 | 0.001 | 0.07 | 0.438 | -0.04 | 0.472 | -0.02 |
| 82 | cg15426878 | 3 : 50368947 | TUSC2 | 0.001 | 0.02 | 0.017 | 0.02 | 0.020 | -0.05 | 0.393 | -0.01 |
| 83 | cg05357287 | 11 : 67408159 | TBX10 | 0.001 | 0.07 | 0.020 | 0.08 | 0.843 | -0.02 | 0.246 | -0.05 |
| 84 | cg14868466 | 10 : 134018073 | DPYSL4, STK32C | 0.001 | -0.02 | 0.005 | -0.03 | 0.035 | 0.05 | 0.026 | 0.03 |
| 85 | cg23205676 | 1 : 17046897 | NBPF1, MST1P9 | 0.001 | 0.06 | 0.212 | 0.04 | 0.263 | -0.08 | 0.005 | -0.10 |
| 86 | cg11099375 | 17 : 62141556 | ICAM2, ERN1 | 0.001 | 0.06 | 0.006 | 0.08 | 0.017 | -0.15 | 7.27 × 10 ^-6^ | -0.13 |
| 87 | cg07617759 | 2 : 33050475 | LTBP1, TTC27 | 0.001 | 0.10 | 0.564 | 0.03 | 0.527 | 0.07 | 0.431 | -0.04 |
| 88 | cg18524934 | 2 : 97164073 | NEURL3, NCAPH | 0.001 | 0.07 | 0.078 | 0.06 | 0.106 | -0.11 | 0.002 | -0.11 |
| 89 | cg21672450 | 12 : 132663793 | NOC4L, GALNT9 | 0.001 | 0.06 | 0.022 | 0.07 | 0.245 | -0.07 | 0.003 | -0.10 |
| 90 | cg26343667 | 3 : 156391915 | TIPARP | 0.001 | 0.03 | 0.076 | 0.03 | 0.278 | -0.04 | 0.333 | -0.02 |
| 91 | cg11516031 | 4 : 15776675 | CD38 | 0.001 | 0.02 | 0.072 | 0.02 | 0.015 | -0.05 | 0.004 | -0.03 |
| 92 | cg07813851 | 1 : 7466976 | VAMP3, CAMTA1 | 0.001 | 0.05 | 0.060 | 0.05 | 0.680 | 0.02 | 0.352 | -0.03 |
| 93 | cg27207776 | 16 : 71393801 | CALB2, ZNF23 | 0.001 | 0.06 | 0.082 | 0.05 | 0.069 | -0.11 | 0.233 | -0.04 |
| 94 | cg27028750 | 5 : 1349422 | CLPTM1L | 0.001 | 0.06 | 0.119 | 0.04 | 0.147 | -0.09 | 9.49 × 10 ^-4^ | -0.11 |
| 95 | cg13173415 | 11 : 12065669 | MICAL2, DKK3 | 0.001 | 0.05 | 0.065 | 0.05 | 0.111 | -0.09 | 0.018 | -0.07 |
| 96 | cg14186846 | 6 : 15256907 | JARID2, DTNBP1 | 0.001 | 0.07 | 0.091 | 0.06 | 0.050 | -0.15 | 0.027 | -0.09 |
| 97 | cg26053073 | 8 : 131455383 | ASAP1 | 0.001 | 0.07 | 0.022 | 0.08 | 0.212 | -0.12 | 0.195 | -0.07 |
| 98 | cg24134918 | 6 : 149886976 | C6orf72 | 0.001 | 0.05 | 0.009 | 0.06 | 0.017 | -0.11 | 0.296 | -0.03 |
| 99 | cg03511957 | 1 : 167765211 | MPZL1, ADCY10 | 0.001 | 0.08 | 0.013 | 0.10 | 0.020 | -0.18 | 0.038 | -0.09 |
| 100 | cg23330788 | 4 : 21699988 | KCNIP4 | 0.001 | 0.07 | 0.225 | 0.05 | 0.999 | 0.00 | 0.644 | -0.02 |

**Supplementary Figure 1. Associations of DNA methylation with exposure and outcome measures were highly correlated comparing sex-regressed and covariate free models.** Effect sizes of the 100 top-ranked DMPs associated with **(a)** exposure group (> 6 months vs < 6 months) **(b)** quantitative measures of exposure time **(c)** IQ at age 15 and **(d)** Theory of Mind at age 11 in covariate-free tests are correlated almost perfectly effect sizes in sex-regressed models at the same 100 probes (*r* = 1.00, *P* ≤ 1.00 × 10^-50^ for each test). The top ten associated sites for the covariate-free model are highlighted in red in each of the panels.

**Supplementary Figure 2. The four top-ranked DMPs associated with severe early-life adversity.** Associations were identified comparing the >6 months and <6 months exposure groups. **(a)** cg11634248, *P* = 2.35 × 10^-5^, **(b)** cg14272935, *P* = 6.89 × 10^-5^, **(c)** cg16668903, *P* = 9.75 × 10^-5^, **(d)** cg06969206, *P* = 1.08 × 10^-4^.

**Supplementary Figure 3. Correlations between the top 100 exposure-associated DMPs and continuous exposure duration.** For the 100 top ranked exposure-associated DMPs (see **Supplementary Table 4**) effect sizes for association with exposure group correlated significantly with effect sizes for association with continuous exposure duration (*r* = 0.93, *P* = 3.03 × 10^-44^). The top ten DMPs associated with exposure group (see **Table 1**) are highlighted in red.

**Supplementary Figure 4. A DMR on chromosome 10 spanning nine sequential 450K array probes (chr10:135340445-135341026) was identified by *comb-P.*** DNA methylation across this region is significantly elevated (*P* = 2.21 × 10^-10^; corrected Šidák *P* = 2.98 × 10^-5^) in individuals exposed to severe institutional deprivation. The >6 months institutionalized group (green) is characterized by consistent hypermethylation across the whole DMR compared to the <6 months institutionalized group (blue) and UK control group (red). An extended ~3kb region spanning the first two exons of *CYP2E1* is shown with the DMR region highlighted in light blue. The probe found to be associated with prenatal maternal antidepressant exposure in neonates by Gurnot *et al* (2015) is highlighted.


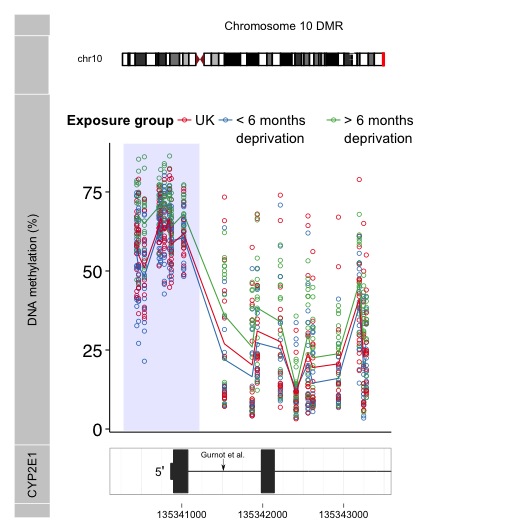


**Supplementary Figure 5. DNA methylation values for CpG sites within the *CYP2E1* DMR quantified using the Illumina 450K array were validated using bisulfite-pyrosequencing.** The assay spanned three CpG sites (cg14250048, cg00436603, and cg01465364) within the DMR, for which there was a highly-significant correlation between DNA methylation levels independently derived from the 450K array and bisulfite-pyrosequencing experiments (*r* = 0.52, *P* = 0.001). Shown is the average DNA methylation (%) across the three CpG sites as measured by bisulfite-pyrosequencing and the 450K array for each of the 36 individuals profiled by both methodologies.

**Supplementary Figure 6. Correlations between the top 100 exposure-associated DMPs and IQ at age 15.** For the 100 top ranked exposure-associated DMPs (see **Supplementary Table 4**) effect sizes for association with exposure group correlated significantly with effect sizes for association with IQ at age 15 (*r* = -0.82, *P* = 4.48 × 10^-25^). The top ten DMPs associated with exposure group (see **Table 1**) are highlighted in red.
